# Consuming Tree Nuts Daily as Between-Meal Snacks Reduces Food Cravings and Improves Diet Quality in American Young Adults at High Metabolic Syndrome Risk

**DOI:** 10.3390/nu17233778

**Published:** 2025-12-02

**Authors:** Kate Lillegard, Annaliese Widmer, John R. Koethe, Heidi J. Silver

**Affiliations:** 1 Division of Gastroenterology, Hepatology and Nutrition, Department of Medicine, Vanderbilt University Medical Center, Nashville, TN 37232, USA; kate.lillegard@vumc.org (K.L.); annaliese.widmer@vumc.org (A.W.); 2 Division of Infectious Diseases, Department of Medicine, Vanderbilt University Medical Center, Nashville, TN 37232, USA; john.r.koethe@vumc.org; 3 Department of Veterans Affairs, Tennessee Valley Healthcare System, Nashville, TN 37212, USA

**Keywords:** metabolic syndrome, young adults, tree nuts, food cravings, diet quality

## Abstract

**Background:** Daily energy intake from snacking behaviors has increased over the past few decades, during which the prevalence of obesity and metabolic syndrome has risen to epidemic proportions. There remains considerable room for improvement in the overall quality of dietary intakes of the U.S. population when compared to national recommendations. Food cravings may contribute to the types of snacks chosen for consumption, and thus, the frequency of foods and food groups consumed, and the overall nutritional quality of the diet. **Methods:** Eighty-four young (28.5 ± 4.3 years) adults with at least one metabolic syndrome risk factor participated in a parallel-arm single-blind randomized trial designed to compare effects of consuming a mix of tree nuts versus typical high-carbohydrate food items as between-meal snacks for 16 weeks. Cravings for 28 common foods via the Food Craving Inventory, short-term dietary intakes via 24 h multi-pass methodology, food group frequency via the Rapid Eating Assessment for Participants, usual hunger and fullness via visual analog scales, appetite-regulating hormones, and diet quality via the Healthy Eating Index—2015 were measured at baseline and end of study. **Results:** Participants in the TNsnack group had significant decreases in cravings for high sweet items and fast-food items, which were associated with decreased frequency of desserts and salty foods along with increased intake of higher protein items. In contrast, no significant reductions in food cravings or preference for sweets were observed in the CHOsnack group. Decreased cravings for sweets by TNsnack participants were associated with increased total GLP-1 levels: cake (r = −0.35, *p* = 0.03), brownies (r = −0.44, *p* = 0.02), candy (r = −0.36, *p* = 0.03) and ice cream (r = −0.33, *p* = 0.04). Overall, the total diet quality score improved by 19% among TNsnack participants. **Conclusions:** Replacing more typical between-meal snacks with tree nuts may reduce food cravings, particularly for sweeter food items that are likely to be nutrient poor and energy dense. By reducing cravings and frequency of intake, consuming tree nuts as snacks could facilitate having a higher quality, more nutrient-dense diet and mitigate potential negative effects of snacking on metabolic health in young adults.

## 1. Introduction

Snacking between meals is a common eating behavior; it has become a global public health concern as snacking often provides a source of excess energy intake contributing to the development of high adiposity (i.e., obesity) with cookies, brownies, ice cream, cake, pies, and candy being the most frequently consumed snacks [1]. Notably, diet assessment in younger adults shows that while daily energy intake from main meals has remained constant, daily energy intake from snacks has increased by 261 kilocalories (kcals) in men and 160 kcals in women over the past three decades [2]. Furthermore, typical high simple carbohydrate and high saturated fat snacks contribute little to the overall nutritional quality of the diet. 

In contrast, more nutrient-rich snack choices may improve diet quality [3]. Indeed, data from several years of National Health and Nutrition Examination Surveys (NHANES) showed that participants who consumed tree nuts as their primary snack choice had higher intakes of Vitamin A, E, C, folate, calcium, iron, magnesium, zinc, potassium, and fiber [4]. These findings were supported by a modeling study using NHANES data that demonstrated replacing typical snacks with tree nuts could reduce energy, saturated fat, added sugars, and sodium intakes while increasing mono- and poly-unsaturated fats, fiber, magnesium, and potassium intakes. Moreover, NHANES data showed choosing tree nuts for snacking yielded higher Healthy Eating Index (HEI) scores, indicating overall improved diet quality [5,6].

A potential non-homeostatic determinant of dietary intake that may adversely affect the nutritional quality of the diet is food cravings [7]. Food cravings, defined as an intense desire to eat specific food items [8], are driven by interactions among brain reward circuits, hormones such as ghrelin, leptin, and GLP-1, and learned associations between thoughts about palatable foods and consumption [9]. Food cravings are common occurring weekly in over two-thirds of young adults [10,11]. Craved foods are typically energy-dense, high in saturated fats and added sugars, and are most frequently consumed as between-meal snacks [12]. While the intensity of food craving is associated with more frequent thoughts about snacks and greater snack consumption, people who have more frequent or more intense food cravings tend to have lower diet quality despite more frequent daily eating occasions [13]. Thus, food cravings, the daily consumption of between-meal snacks, and the frequency of foods consumed are highly interconnected in everyday life. 

The relationship between food frequency and indices of diet quality have been investigated in several studies which have demonstrated moderate to strong reproducibility and validity when using food frequency questionnaires to assess measurements of diet quality [14,15,16,17]. Based on frequency of consumption of various foods and food groups, the U.S. Department of Agriculture’s HEI has been used to measure diet quality in numerous surveillance, epidemiologic, and intervention studies since 1995 as it is designed to determine alignment of dietary intake with the U.S. Dietary Guidelines for Americans [18]. Importantly, low diet quality scores significantly associate with multiple cardiometabolic disease outcomes including metabolic syndrome as well as type 2 diabetes, cardiovascular diseases, and all-cause mortality [19,20,21].

Although observational and modelling studies suggest the potential benefits of tree nut consumption, randomized controlled trials investigating the effects of tree nuts as between-meal snacks on food cravings and diet quality remain lacking. The purpose of the present study is to determine relationships between changes in food cravings, frequency of food items consumed, and diet quality in young adults enrolled in a randomized trial testing consumption of tree nuts as between-meal snacks versus more typical high-carbohydrate snacks. We hypothesized that participants in the tree nuts snack group would have reduced cravings for salty and sweet foods that would be associated with reduced frequency of those food items, and thus, improved diet quality. 

## 2. Materials and Methods

### 2.1. Participants

Participants were recruited and enrolled from December 2019 through January 2023 via ResearchMatch.org, the Vanderbilt University Medical Center research list-serve email, and study flyers posted around the greater Nashville, Tennessee metropolitan area. Eligibility criteria included age 22–36 years, weight-stability (defined as self-reported weight maintenance within 3 pounds for 3 months prior to enrollment), body mass index (BMI) of 24.5 to 34.9 kg/m^2^, and having at least one of the five metabolic syndrome (MetSx) risk factor criteria, as previously described [22]. Exclusion criteria were tree nut allergy, chronic disease diagnosis (diabetes mellitus, liver disease, renal insufficiency, cardiovascular disease, active malignancy, polycystic ovary syndrome, inflammatory bowel disease, chronic respiratory disease, or celiac disease), prescribed medications for dyslipidemia or hyperglycemia, pregnancy, lactation, or current narcotics usage, smoking, vaping, or excessive alcohol intake. All participants provided written informed consent, study methods were conducted in compliance with the 1964 Declaration of Helsinki and approved by the VUMC Institutional Review Board (IRB#190222 approved 10 January 2019), and the study is registered at ClinicalTrials.gov (NCT03969264). As previously reported, power analysis estimated 50 completers per snack group would provide 80% probability to detect a clinically meaningful difference in primary and secondary outcomes [23]. 

### 2.2. Study Design and Intervention

Full details of the study protocol have been previously reported [23,24]. The dietary intervention was a 16-week, prospective, parallel-arm, single-blind, randomized trial wherein participants first underwent a 2-week run-in of consuming a standardized eucaloric diet for weight maintenance that included high-carbohydrate between-meal snacks and no nut products. At the completion of week 2, a permuted block randomization scheme was employed to stratify participants by BMI and assign them to daily snacks as tree nuts (TNsnack) or high-carbohydrate items (CHOsnack) for 16 weeks. Snacks were consumed twice daily as a component of 7-day cycle menus that were developed using Nutrition Data System for Research (NDSR v.2020) software. Menus were designed to provide 3 meals and 2 snack times daily based on the Institute of Medicine acceptable distribution ranges for macronutrients (25–35% fat, 45–55% carbohydrate, 15–20% protein). All menu items excluded peanuts, tree nuts, and nut butters. Snack calories were 15–20% of total daily energy needs determined individually from measured resting energy expenditure (ParvoMedics TrueOne 2400, Salt Lake City, UT, USA) multiplied by a physical activity factor to ensure weight maintenance. Tree nuts snacks consisted of a 33.5 g mix of unsalted raw almonds, walnuts, pecans, macadamia nuts, hazel nuts, pistachios, and cashews. Carbohydrate snacks included unsalted pretzels, animal crackers, graham crackers, and Nutrigrain/granola-type bars. Tree nuts and carbohydrate snacks were comparable in energy (kcal), protein, fiber, and sodium content. All consumption occurred between 6:00 a.m. and 6:00 p.m. Participants met with trained Registered Dietitians biweekly to receive nutrition counseling and recommendations aligned with the 2020–2025 Dietary Guidelines along with 2-week supplies of the pre-weighed and portioned snacks. Anthropometrics, blood draws, metabolic testing, and all questionnaires including the Food Craving Inventory and Healthy Eating Index were administered at baseline, week 2, and week 18 at the Vanderbilt Center for Human Nutrition.

### 2.3. Dietary Intakes and Adherence

Dietary intake within the 24 h prior to a study visit was assessed using NDSR software by employing the validated USDA multi-pass diet recall methodology with a standardized script and measuring utensils (spoons, cups, bowls, plates) of varying sizes to prompt reliable estimation of portion sizes consumed. Frequency of intakes were estimated using the Rapid Eating Assessment for Participants (REAP) which has shown high test–retest reliability in querying the frequency of consumption of food items and food groups during the prior week [25,26]. Diet adherence was assessed from diet recall data, food intake logs reviewed at dietitian visits, 24 h urinary urea output [23], and plasma fatty acid analysis [24].

### 2.4. Food Cravings

The Food Craving Inventory (FCI) measures the frequency of cravings for 28 specific food items with 8 items in the high fat foods subscale, 8 items in the high sweet foods subscale, 8 items in the high starch foods subscale, and 4 items in the fast foods subscale. For each of the 28 items, response to the query “over the past month, how often have you experienced a craving for…” is rated as 1 = never, 2 = rarely, 3 = sometimes, 4 = often, and 5 = always/almost every day. Content, concurrent, construct, and discriminant validity have been reported [27].

### 2.5. Diet Quality

The Healthy Eating Index (HEI) is considered an indicator of diet quality as it determines whether food intake aligns with the Dietary Guidelines for Americans. Construct validity, reliability, and criterion validity have been published [28]. Total diet quality score and scores for the 13 components of HEI-2015 were acquired from NDSR output files along with the number of servings in cup equivalents for dairy, fruits, vegetables, proteins, and grains. A weighted approach that utilizes densities instead of amounts enables calculation of a total HEI-2015 score that ranges from 0 to 100 [29].

### 2.6. Clinical Biomarkers

Venous whole blood samples were collected in the fasting state. Prior to centrifugation, samples were kept on ice and preservatives were added to ghrelin and glucagon to prevent enzymatic breakdown. Plasma was then aliquoted into 2 mL portions, stored at −80 °C, and submitted monthly in batches to the Vanderbilt University Medical Center Hormone Assay & Analytical Services Core Laboratory for enzymatic assay of plasma glucose with detection 20–900 mg/dL and radioimmunoassay (RIA) of plasma insulin, total and active ghrelin, total and active glucagon, and adiponectin. All assays were performed in duplicate with inter-assay CVs ≤ 10%. 

### 2.7. Statistical Analysis

Data were assessed for normality visually using histograms and Q-Q plots. We employed Wilcoxon signed-rank tests to assess within-group changes for TNsnack and CHOsnack groups and general linear models for between-group changes. Cohen’s d values enabled determining the magnitude of the effect for HEI variables, as the total HEI score denotes the primary outcome of diet quality. Linear regression was used to predict total HEI score with participant sex, age, and baseline BMI as covariates. Spearman’s rho correlation coefficients were calculated to evaluate associations between changes in food cravings, food frequencies, and total HEI score within each group. As the correlation tests were preplanned and the number of tests small, there was no need to adjust for multiple comparisons [30]. SPSS version 31.0 (IBM, Armonk, NY, USA) and R version 4.1.2 (R Foundation for Statistical Computing, Vienna, Austria) were used for analyses. 

## 3. Results

The intervention was completed by 40 participants in the TNsnack group and 44 participants in the CHOsnack group. Five participants dropped out from each snack group: 1 due to pregnancy, 2 due to weight loss, and 7 were lost to follow-up [23]. The mean age of the 84 participants who completed the study was 28.5 ± 4.3 years and mean BMI was 28.4 ± 3.7 kg/m^2^. At baseline, 25% of participants had 2 of the MetSx risk factors and 10.7% had 3 or more risk factors. No significant differences were observed between TNsnack and CHOsnack groups for age, sex, BMI, physical activity level, or MetSx risk score. There were also no significant differences at baseline between groups for daily averages of total amount of food consumed (3475.1 ± 1349.2 g/day), energy intake (2024.1 ± 727.1 kcals/day), or macronutrient distribution of the diet (fat %kcals: 37.6 ± 8.8; carbohydrate %kcals: 43.3 ± 11.8; protein %kcals: 18.0 ± 6.4) or micronutrient intake (Supplemental Table S1).

At completion of the 16 week intervention period, participants in the TNsnack group reported significantly reduced frequency in cravings for pizza (−0.33 ± 0.57, *p* < 0.001), cookies (−0.63 ± 1.03, *p* < 0.001), brownies (−0.45 ± 0.71, *p* < 0.001), donuts (−0.40 ± 0.90, *p* = 0.008), candy (−0.33 ± 0.92, *p* = 0.03), ice cream (−0.55 ± 1.08, *p* = 0.003) and chips (−0.33 ± 1.03, *p* = 0.05) (Table 1). The reduction in cravings for high sugar items was associated with a 12.5% decrease in the proportion of TNsnack participants who reported a preference for sweet taste (r = 0.30, *p* = 0.03). Consequently, the TNsnack group had significant reductions in the total FCI subscales for high sweet items (−2.90 ± 4.72, *p* < 0.001) and fast-food items (−1.15 ± 2.09, *p* = 0.001) whereas no significant changes in cravings for specific items, taste preference, or FCI subscales were observed in the CHOsnack group.

In addition to the reductions in food cravings reported by TNsnack participants, there was a significant reduction in the frequency of consuming frozen desserts (−0.18 ± 0.64, *p* = 0.04) and salty snacks (−0.41 ± 0.88, *p* = 0.006) along with increased frequency in daily servings (svgs) of high protein food items (5.10 ± 0.92 svgs/day, *p* < 0.001) including increased frequency of consuming higher protein items from seafood and plant sources (4.37 ± 3.92 svgs/day, *p* < 0.001) (Table 2). The only significant change in food item frequency by CHOsnack participants was a decrease in daily fruit intake (−0.36 ± 0.89 svgs/day, *p* = 0.01).

No significant changes were observed in plasma glucose or insulin levels in either snack group. There was a significant rise in average total ghrelin and total GLP-1 levels at 16 weeks in TNsnack participants. Increased total GLP-1 was associated with decreased cravings for cake (r = −0.35, *p* = 0.03), brownies (r = −0.44, *p* = 0.02), candy (r = −0.36, *p* = 0.03) and ice cream (r = −0.33, *p* = 0.04). Still, ratings of hunger and fullness did not change in the TNsnack group (Table 3). While TNsnack participants reported a significant decrease in the total amount of food consumed daily (−468.12 ± 1035.93 g/day, *p* = 0.007), there was no change in their energy intake (148 ± 787.02 kcals/day, *p* = 0.24), and thus, no change in body weight. Conversely, there was a trend toward increased hunger and reduced fullness ratings by the CHOsnack group and CHOsnack participants had significantly increased energy intakes (349.02 ± 833.82 kcals/day, *p* = 0.008) with average body weight increasing by 0.78 ± 1.95 kg at week 18 (*p* = 0.01).

In the TNsnack group, total HEI score (Table 4) was significantly increased (10.01 ± 15.16, *p* < 0.001, *d* = 0.7), which reflected increases in the HEI categories of high protein food items (0.70 ± 1.71, *p* = 0.01, *d* = 0.4) and unsaturated fatty acids (4.33 ± 4.09, *p* < 0.001, *d* = 0.3). Unexpectedly, a decrease was observed in the HEI category of dairy food items (−1.57 ± 4.93, *p* = 0.05, *d* = 0.3). The increase in total HEI score was significantly associated with the reduction in food cravings for brownies (r = 0.31, *p* = 0.04), donuts (r = −0.37, *p* = 0.02) and candy (r = 0.35, *p* = 0.03), and the increased frequency of higher protein food servings (r = 0.51, *p* < 0.001). Multivariable linear regression showed that 50% of the variance in total HEI score was explained by the increased intake of tree nuts, mono- and poly-unsaturated fats, and high protein servings with the reduction in frequency of salty snacks and total sodium intake when controlling for sex, age, and baseline BMI (F = 6.36, *p* < 0.001). In contrast to the improvements in the HEI indicators of diet quality observed in the TNsnack group, no changes in total HEI score or HEI categories were observed in the CHOsnack group.

## 4. Discussion

The principal finding of this study is replacing typical high-carbohydrate between-meal snacks with a mixture of tree nuts reduced food cravings and frequency of consumption of high simple carbohydrate and fast-food items while improving overall diet quality in young adults at risk for metabolic syndrome. Consumption of snacks is often considered a major contributor to the development of obesity and cardiometabolic disease as the category of “snack foods” is typically comprised of items that are high in saturated fats, simple sugars, and low in nutritional quality. In contrast, several studies show no adverse effects of consuming tree nuts on energy balance and body weight; it is likely that calories from tree nuts snack consumption are compensated for by reduced subsequent energy intake [3,31]. Moreover, our prior findings [23] showed substituting tree nuts for typical high-carbohydrate snacks reduced metabolic syndrome risk in young adults which is consistent with evidence from a meta-analysis of feeding trials that indicates tree nut consumption may reduce weight, BMI, and waist circumference—a primary risk factor for the development of the metabolic syndrome [32]. 

While prior investigation of the impact of tree nuts as snacks on measured indices of diet quality is lacking, NHANES data employing the HEI with >11,000 adults suggests that snacking can improve diet quality [4]. Indeed, NHANES data indicates that improved diet quality of the U.S. population over the past two decades has been driven by increased consumption of whole grains, poultry, and nuts [33]. In our study, the increased intake of unsaturated fat from tree nuts and the higher protein content of items consumed were the most significant contributors to the improvement observed in diet quality in TNsnack participants. Whereas the increase in HEI score in the general U.S. population has been small (~4%), the change in the present study was large enough in magnitude (19%) to be considered a clinically meaningful improvement in diet quality. 

Whether the dietary protein or fat content of tree nuts directly influences appetite-related sensations is uncertain. While evidence of differential effects on hunger and fullness based on the composition (degree of saturation, chain length, and position of double bonds) of fatty acids remains inconclusive, many studies indicate protein is the most potent macronutrient with regard to satiety effects, and thus, higher protein intake could mediate both subjective appetite responses and appetite-regulating gastrointestinal hormones. Further, tree nuts are a rich source of other bioactive compounds including soluble and insoluble fibers, which may also have appetite-modulating effects [34,35]. Notably, a meta-analysis of 31 trials [35] and dietary intervention studies replacing high-carbohydrate snacks with almonds [36] suggest that the consumption of mixed tree nuts as snacks would suppress hunger and desire to eat. However, no reductions in hunger, fullness, energy intakes, or body weight were observed in the TNsnack group. In contrast, there was a trend toward increased hunger in the CHOsnack participants who also presented with increased energy intakes and body weight. 

Among the mechanisms by which tree nuts may affect appetite and frequency of food intake are changes in the circulating concentrations of gut-derived hormones such as ghrelin and GLP-1 which are stimulated by ingestion of dietary fat, carbohydrate, and protein. While all three macronutrients affect stomach secretion of ghrelin similarly, the intestinal secretion of GLP-1 is primarily driven by higher protein intake. The opposing effects of these two hormones on vagal afferent pathways interact to regulate eating behavior and food intake [37]. The increase in endogenous GLP-1 may have suppressed appetite and decreased motivation to consume higher calorie foods. This may explain why the increase in ghrelin levels observed in the TNsnack group, which would be expected to increase appetite and food intake, did not affect hunger, energy intake, or body weight in TNsnack participants. Further, it appears that the magnitude of the increase in the active form of ghrelin was inadequate for stimulating hunger and appetite [38]. It is also plausible that the increase in total ghrelin levels observed in the TNsnack group reflects the ability of bioactive components of tree nuts to restore impaired efficiency from being in a state of metabolic dysregulation [39].

Beyond hunger, higher ghrelin levels have been associated with increased food cravings [40]. Yet, consuming tree nuts as snacks also resulted in reductions in self-reported food cravings, specifically for sweet items and items typically considered fast foods. While food cravings can be for a specific food item, they can also be for a group of foods. Sweet foods are the most commonly reported craved foods, possibly due to our innate preference or liking for sweet taste [41,42]. After 16 weeks of intervention, 12.5% fewer TNsnack participants reported a preference for sweet taste. This finding is consistent with evidence that real-life sensory and environmental experiences can modify sweet preference [43]. Studies reveal that food cravings predict eating behavior, even when people already feel satiated [44]. The reduction in food cravings along with diminished preference for sweet taste in TNsnack participants appears to have triggered less frequent intake of frozen desserts and sugary snacks—which contributes to improved overall diet quality.

Overall, unlike the CHOsnack group, participants in the TNsnack group experienced a substantial reduction in food cravings, consumption of food items that are frequently craved, and increase in total HEI score, indicating dietary intakes became better aligned with recommendations from the 2015–2020 Dietary Guidelines for Americans—guidelines that promote a healthier dietary pattern with limited intake of saturated fats, added sugars, and sodium along with increased intake of unsaturated fats and high protein foods [45]. Despite few studies, both dietary patterns and improved diet quality have been associated with reduced risk for MetSx [46], possibly as a means of altering the specific modifiable cardiometabolic risk factors that comprise the metabolic syndrome [47]. Importantly, development of a more internationally suitable metric of diet quality, the global diet quality score (GDQS), has also shown a positive relationship between higher diet quality and reduced MetSx risk [48].

The limitations of this study include that assessment of food cravings, 24 h dietary intakes, and frequency of food consumption are subject to measurement error due to recall or reporting bias. The use of visual analog scales to assess hunger and fullness also relies on self-report rather than more objective modalities. Additionally, the sample size of each group limits the ability to observe statistically significant differences in factors that have large variability. However, the strengths of the study include the randomized parallel arm design, the incorporation of a run-in period standardizing dietary intakes for weight maintenance, investigation of a real-world pragmatic dietary snack intervention, and the engagement of trained research dietitians employing a validated multiple-pass diet assessment technique, standardized prompts, and usage of visual aids such as measuring utensils for estimation of food portion sizes thereby mitigating potential recall error. 

## 5. Conclusions

In conclusion, replacing more typical between-meal snacks with tree nuts may reduce food cravings, particularly for sweeter food items that are likely to be nutrient poor and energy dense. By reducing cravings and the frequency of intake, substituting tree nuts for high-carbohydrate snacks could facilitate having a higher quality more nutrient-dense diet and mitigate potential negative effects of snacking on metabolic health of young adults. Indeed, adopting the substitution of a single food item or group is a relatively simple real-world strategy that can be incorporated in dietary guidelines and health promotion programs that aim to improve cardiometabolic health. Nevertheless, the changes and trends observed require larger samples for longer periods of time to fully determine generalizability to the general population of young adults and to determine sustainability of the effects observed. The effects of specific types of tree nuts, changes in brain activation, and impact on other population subgroups might also be considered in future investigations.

## Figures and Tables

**Table 1:** Changes in food craving scores over a 16-week intervention period.

	Tree Nuts Snack Group (N = 40)			High-Carbohydrate Snack Group (N = 44)		
	Baseline	End of Study	*p*-Value	Baseline	End of Study	*p*-Value
Cake	0.90 ± 0.78	0.75 ± 0.67	0.29	0.98 ± 0.94	0.86 ± 0.83	0.45
Cookies	1.78 ± 1.17	1.15 ± 0.92	<0.001	1.38 ± 1.17	1.21 ± 1.09	0.28
Brownies	0.95 ± 0.85	0.50 ± 0.72	<0.001	1.09 ± 1.02	1.07 ± 1.23	0.88
Ice Cream	1.73 ± 1.04	1.18 ± 1.13	0.003	1.58 ± 1.20	1.33 ± 1.16	0.19
Candy	1.00 ± 0.96	0.68 ± 0.97	0.03	1.05 ± 1.15	1.16 ± 1.04	0.49
Chocolate	1.78 ± 1.29	1.55 ± 1.13	0.25	1.63 ±1.22	1.63 ± 1.00	0.99
Cinnamon Rolls	0.63 ± 0.74	0.45 ± 0.64	0.18	0.84 ± 0.92	0.53 ± 0.88	0.07
Donuts	0.88 ± 0.85	0.48 ± 0.64	0.008	0.90 ± 0.92	0.98 ± 0.98	0.62
High Sweets Subscale	9.7 ± 4.5	6.7 ± 4.7	<0.001	9.9 ± 5.0	8.8 ± 5.3	0.20
Chips	1.64 ± 1.27	1.31 ± 1.17	0.05	1.60 ± 1.11	1.63 ± 1.05	0.88
Hamburger	1.50 ± 1.04	1.25 ± 1.10	0.11	1.09 ± 1.06	1.16 ± 1.09	0.66
French Fries	1.75 ± 1.13	1.55 ± 1.13	0.12	1.49 ± 1.12	1.70 ± 1.15	0.14
Pizza	1.80 ± 0.79	1.48 ± 0.75	<0.001	1.79 ± 1.04	1.51 ± 0.96	0.06
Fast Food Subscale	6.7 ± 3.0	5.5 ± 3.0	0.001	6.0 ± 3.0	6.0 ± 3.2	0.95
Pancakes/Waffles	0.95 ± 0.78	0.78 ± 0.77	0.21	1.11 ± 1.14	0.79 ± 0.97	0.06
Cereal	0.85 ± 0.95	0.70 ± 0.97	0.36	0.86 ± 1.21	0.67 ± 0.92	0.24
Sandwich Bread	0.80 ± 1.02	0.80 ± 1.07	0.99	0.72 ± 0.98	0.79 ± 0.97	0.61
Baked Potato	1.03 ± 1.19	0.83 ± 1.06	0.10	0.58 ± 0.91	0.49 ± 0.88	0.49
Rolls	0.60 ± 1.01	0.55 ± 1.04	0.64	0.53 ± 0.93	0.67 ± 1.13	0.29
Biscuits	0.58 ± 0.71	0.40 ± 0.59	0.16	1.07 ± 1.16	0.84 ± 1.11	0.14
Pasta	1.53 ± 1.13	1.58 ± 1.01	0.77	1.27 ± 1.18	1.05 ± 0.89	0.21
Rice	1.10 ± 1.15	1.03 ± 1.05	0.62	0.93 ± 1.18	1.21 ± 1.12	0.08
High Starch Subscale	6.9 ± 5.4	6.7 ± 3.0	0.67	6.4 ± 4.5	6.5 ± 4.6	0.82
Corn Bread	0.35 ± 0.62	0.23 ± 0.48	0.13	0.33 ± 0.52	0.40 ± 0.79	0.54
Fried Chicken	1.13 ± 1.04	1.05 ± 1.06	0.61	1.26 ± 1.18	1.07 ± 1.08	0.24
Fried Fish	0.58 ± 0.68	0.50 ± 0.75	0.56	0.51 ± 0.77	0.53 ± 0.85	0.81
Bacon	1.25 ± 1.10	1.33 ± 1.16	0.64	1.23 ± 1.25	1.11 ± 1.26	0.40
Hot Dog	0.63 ± 0.87	0.33 ± 0.62	0.006	0.56 ± 0.77	0.70 ± 0.99	0.36
Sausage	0.58 ± 0.87	0.75 ± 1.01	0.19	0.88 ± 0.98	0.84 ± 1.19	0.77
Steak	1.15 ± 1.17	1.15 ± 1.08	0.99	1.21 ± 1.26	1.21 ± 1.25	0.99
Gravy	0.23 ± 0.54	0.28 ± 0.56	0.59	0.47 ± 0.91	0.40 ± 0.76	0.45
High Fats Subscale	5.8 ± 4.4	5.4 ± 4.2	0.48	6.4 ± 4.7	6.3 ± 5.7	0.74

Data are presented as mean ± standard deviation. Food items within subscales are based on factor loading of the Food Craving Inventory developed by White et al., 2002 [27].

**Table 2:** Changes in frequency of consumption by food group or food group servings over a 16-week intervention period.

	TN Snack Group (N = 40)			CHO Snack Group (N = 44)		
	Baseline	End of Study	*p*-Value	Baseline	End of Study	*p*-Value
Frequency of Consumption of Item/Food Group in an Average Week *						
Salted Snacks	2.28 ± 0.72	1.87 ± 0.86	0.006	2.16 ± 1.06	2.27 ± 0.95	0.28
Sugary Snacks	2.28 ± 1.05	1.95 ± 0.92	0.05	2.25 ± 1.16	2.23 ± 1.03	0.87
Frozen Desserts	1.46 ± 0.91	1.28 ± 0.69	0.04	1.50 ± 0.93	1.45 ± 0.90	0.70
Sugar Sweetened Beverages	0.93 ± 1.00	0.85 ± 0.98	0.65	1.07 ± 1.21	1.00 ± 1.20	0.55
Non-Caloric Beverages	0.85 ±1.25	0.85 ± 1.23	0.99	1.39 ± 1.45	1.39 ± 1.42	0.99
Non-Nutritive Sweeteners	1.08 ± 1.16	1.15 ± 1.19	0.29	1.39 ± 1.22	1.36 ± 1.13	0.87
Homemade Meals	3.26 ± 0.79	3.03 ± 1.01	0.04	3.25 ± 0.81	3.07 ± 0.93	0.04
Restaurant Meals	1.95 ± 0.65	1.92 ± 0.70	0.41	1.98 ± 0.70	1.98 ± 0.63	0.99
Ready-to-Eat Meals	1.59 ± 0.94	1.41 ± 0.84	0.15	1.59 ± 1.04	1.66 ± 0.96	0.62
Fermented Foods	0.72 ± 0.83	0.82 ± 0.85	0.40	1.00 ± 0.86	1.02 ± 0.88	0.82
Extra Virgin Olive Oil	2.69 ± 1.00	2.56 ± 1.05	0.41	2.73 ± 1.13	2.50 ± 1.11	0.08
Water	3.50 ± 0.72	3.45 ± 0.71	0.32	3.25 ± 0.89	3.32 ± 0.96	0.57
Poultry (svgs)	2.77 ± 0.84	2.87 ± 0.66	0.16	2.91 ± 0.71	2.91 ± 0.71	0.99
Meat and Egg (svgs)	3.30 ± 0.72	3.28 ± 0.68	0.40	3.48 ± 0.59	3.34 ± 0.61	0.14
Red Meat (svgs)	1.82 ± 0.82	1.82 ± 0.76	0.50	2.02 ± 0.90	1.95 ± 0.86	0.54
High-Fat Red Meat (svgs)	1.46 ± 0.82	1.33 ± 0.70	0.17	1.50 ± 0.93	1.48 ± 0.79	0.83
Seafood (svgs)	1.46 ± 0.85	1.33 ± 0.93	0.17	1.50 ± 0.85	1.45 ± 0.76	0.62
Dairy (svgs)	2.87 ± 1.13	2.62 ± 1.11	0.13	2.61 ± 1.13	2.39 ± 1.08	0.17
Dairy Substitute (svgs)	1.28 ± 1.36	1.31 ± 1.28	0.85	1.75 ± 1.38	1.34 ± 1.16	0.01
Fruit (svgs)	2.44 ± 1.07	2.51 ± 0.88	0.57	2.59 ± 0.90	2.23 ± 0.94	0.01
Vegetable (svgs)	2.85 ± 0.99	3.10 ± 0.79	0.09	3.00 ± 0.86	2.82 ± 0.92	0.17
Whole Grains (svgs)	2.77 ± 0.93	2.62 ± 1.09	0.11	2.43 ± 1.07	2.55 ± 0.98	0.51
Fruit Servings (cup eq)	0.76 ± 1.68	0.35 ± 0.50	0.13	0.84 ± 1.19	0.51 ± 0.48	0.06
**Frequency of Consumption of Food Group Servings on an Average Day**						
Total Grain Servings (oz eq)	7.27 ± 5.51	5.55 ± 3.41	0.08	6.99 ± 4.46	8.77 ± 4.21	0.06
Whole Grain Servings (oz eq)	1.88 ± 2.39	1.68 ± 2.55	0.69	1.81 ± 2.18	2.57 ± 2.46	0.13
Refined Grain Servings (oz eq)	5.39 ± 5.66	3.86 ± 2.62	0.09	5.18 ± 3.94	6.20 ± 3.62	0.19
Vegetable Servings (cup eq)	1.42 ± 1.14	1.39 ± 1.02	0.89	1.92 ± 1.33	1.99 ± 1.32	0.77
Greens and Beans Servings (cup eq)	0.30 ± 0.49	0.36 ± 0.54	0.62	0.60 ± 0.65	0.68 ± 0.75	0.55
Dairy Servings (cup eq)	1.57 ± 1.41	1.02 ± 0.94	0.03	1.38 ± 1.35	1.41 ± 1.39	0.89
Protein Servings (oz eq)	6.06 ± 4.16	11.16 ± 5.47	<0.001	8.28 ± 5.41	8.22 ± 5.26	0.95
Seafood and Plant Protein Servings (cup eq)	1.30 ± 2.07	5.67 ± 3.41	<0.001	2.88 ± 2.89	1.53 ± 2.50	0.05

* Values are provided as mean ± standard deviation based on the frequency response as: 0 = never, 1 = rarely (less than 1 time/week), 2 = occasionally (1–2 times/week), 3 = regularly (3–5 times/week), or 4 = daily.

**Table 3:** Changes in biomarkers and hedonics related to food intake over a 16-week intervention period.

	Tree Nuts Snack Group (N = 40)			High-Carbohydrate Snack Group (N = 44)		
	Baseline	End of Study	*p*-Value	Baseline	End of Study	*p*-Value
Glucose (mg/dL)	86.3 ± 10.4	85.8 ± 11.9	0.77	82.9 ± 8.2	84.4 ± 8.2	0.42
Insulin (µU/mL)	9.5 ± 9.9	8.1 ± 5.0	0.22	6.8 ± 3.4	7.0 ± 4.1	0.73
Active Ghrelin (pg/mL)	72.1 ± 78.3	92.7 ± 89.3	0.10	89.7 ± 106.7	97.5 ± 100.5	0.41
Total Ghrelin (pg/mL)	439.4 ± 444.3	644.8 ± 613.4	0.04	594.9 ± 571.8	698.5 ± 615.4	0.14
Active GLP-1 (pg/mL)	15.7 ± 11.8	18.3 ± 13.9	0.20	19.9 ± 44.9	21.8 ± 40.9	0.32
Total GLP-1 (pg/mL)	144.2 ± 57.5	160.6 ± 55.7	0.04	136.8 ± 72.7	141.6 ± 67.4	0.52
Adiponectin (mcg/mL)	31.8 ± 18.4	21.6 ± 8.3	<0.001	35.5 ± 33.2	22.6 ± 11.8	<0.001
Hunger	4.9 ± 1.4	4.8 ± 1.6	0.93	4.6 ± 1.8	5.2 ± 1.7	0.07
Fullness	6.1 ± 1.5	6.1 ± 1.6	0.85	6.3 ± 1.3	5.8 ± 1.5	0.10

Data are presented as mean ± standard deviation. Hunger and fullness were assessed using visual analog scales anchored with scores of 0 and 10.

**Table 4:** Changes in diet quality based on Healthy Eating Index (HEI) scores over a 16-week intervention period.

	Tree Nuts Snack Group (N = 40)			High-Carbohydrate Snack Group (N = 44)		
	Baseline	End of Study	*p*-Value	Baseline	End of Study	*p*-Value
HEI Fruits	1.51 ± 1.92	0.98 ± 1.52	0.08	1.90 ± 1.89	1.33 ± 1.31	0.08
HEI Vegetables	2.96 ± 1.80	2.75 ± 1.65	0.53	3.52 ± 1.69	3.14 ± 1.60	0.14
HEI Whole Grains	4.39 ± 4.18	3.73 ± 3.95	0.34	4.27 ± 3.98	5.51 ± 4.00	0.12
HEI Refined Grains	5.94 ± 4.38	7.75 ± 3.09	0.02	6.90 ± 3.58	6.16 ± 3.83	0.34
HEI Dairy	5.17 ± 3.85	3.60 ± 3.06	0.05	4.46 ± 3.68	3.96 ± 3.19	0.45
HEI Protein	4.14 ± 1.51	4.85 ± 0.62	0.01	4.45 ± 1.04	4.44 ± 1.06	0.95
HEI Fatty Acids	4.71 ± 3.90	9.04 ± 2.16	<0.001	6.08 ± 3.91	6.00 ± 3.50	0.89
HEI Sodium	4.11 ± 3.57	6.31 ± 3.50	0.01	3.72 ± 3.57	4.15 ± 3.54	0.56
HEI Added Sugars	8.05 ± 2.83	8.58 ± 2.35	0.30	8.26 ± 2.81	8.72 ± 1.91	0.31
HEI Total Score	51.98 ± 17.06	62.00 ± 12.16	<0.001	58.09 ± 12.94	56.51 ± 12.93	0.57

Data are presented as mean ± standard deviation. The HEI-2015 version was used to assess diet quality: whole grains, dairy, fatty acids, refined grains, sodium, and added sugars have a maximum of 10 points; fruits, vegetables, and protein have a maximum of 5 points; and the HEI-2015 total score has a maximum of 100 points.

## Data Availability

Data described in the manuscript, code book, and analytic code will be made available upon request pending application and approval.

## Supplementary Materials

The following supporting information can be downloaded at https://www.mdpi.com/article/10.3390/nu17233778/s1, Table S1: Changes in Micronutrient Intakes Over a Period of 16 Weeks.

## References

[1] Mazlan N., Horgan G., Whybrow S., Stubbs J. (2006). Effects of increasing increments of fat- and sugar-rich snacks in the diet on energy and macronutrient intake in lean and overweight men. Br. J. Nutr..

[2] Piernas C., Popkin B.M. (2010). Snacking increased among U.S. adults between 1977 and 2006. J Nutr..

[3] Hampl J.S., Heaton C.L., Taylor C.A. (2003). Snacking patterns influence energy and nutrient intakes but not body mass index. J. Hum. Nutr. Diet..

[4] O’Neil C.E., Nicklas T.A., Fulgoni V.L. (2015). Tree nut consumption is associated with better nutrient adequacy and diet quality in adults: National Health and Nutrition Examination Survey 2005–2010. Nutrients.

[5] Zizza C.A., Xu B. (2012). Snacking is associated with overall diet quality among adults. J. Acad. Nutr. Diet..

[6] Rehm C.D., Drewnowski A. (2017). Replacing American snacks with tree nuts increases consumption of key nutrients among US children and adults: Results of an NHANES modeling study. Nutr. J..

[7] Gilhooly C.H., Das S.K., Golden J.K., McCrory M.A., Dallal G.E., Saltzman E., Kramer F.M., Roberts S.B. (2007). Food cravings and energy regulation: The characteristics of craved foods and their relationship with eating behaviors and weight change during 6 months of dietary energy restriction. Int. J. Obes..

[8] Weingarten H.P., Elston D. (1990). The phenomenology of food cravings. Appetite.

[9] Kalon E., Hong J.Y., Tobin C., Schulte T. (2016). Psychological and neurobiological correlates of food addiction. Int. Rev. Neurobiol..

[10] Pelchat N.L. (1997). Food cravings in young and elderly adults. Appetite.

[11] Hill A.J. (2007). The psychology of food craving. Proc. Nutr. Soc..

[12] Richard A., Meule A., Reichenberger J., Blechert J. (2017). Food cravings in everyday life: An EMA study on snack-related thoughts, cravings, and consumption. Appetite.

[13] Taetzsch A., Roberts S.B., Gilhooly C.H., Lichtenstein A.H., Krauss A.J., Bukhari A., Martin E., Hatch-McChesney A., Das S.K. (2020). Food cravings: Associations with dietary intake and metabolic health. Appetite.

[14] Newby P.K., Hu F.B., Rimm E.B., Smith-Warner S.A., Feskanich D., Sampson L., Willett W.C. (2003). Reproducibility and validity of the Diet Quality Index Revised as assessed by use of a food-frequency questionnaire. Am. J. Clin. Nutr..

[15] Troeschel A.N., Hartman T.J., Flanders W.D., Wang Y., Hodge R.A., McCullough L.E., Mitchell D.C., Sampson L., Patel A.V., McCullough M.L. (2020). The American Cancer Society Cancer Prevention Study-3 FFQ has reasonable validity and reproducibility for food groups and a diet quality score. J. Nutr..

[16] Benítez-Arciniega A.A., Mendez M.A., Baena-Díez J.M., Rovira Martori M.A., Soler C., Marrugat J., Covas M.I., Sanz H., Llopis A., Schröder H. (2011). Concurrent and construct validity of Mediterranean diet scores as assessed by an FFQ. Public Health Nutr..

[17] Yue Y., Yuan C., Wang D.D., Wang M., Song M., Shan Z., Hu F., Rosner B., Smith-Warner S.A., Willett W.C. (2022). Reproducibility and validity of diet quality scores derived from food-frequency questionnaires. Am. J. Clin. Nutr..

[18] Kirkpatrick S.I., Reedy J., Krebs-Smith S.M., Pannucci T.E., Subar A.F., Wilson M.M., Lerman J.L., Tooze J.A. (2018). Applications of the Healthy Eating Index for surveillance, epidemiology, and intervention research: Considerations and caveats. J. Acad. Nutr. Diet..

[19] Yuguang L., Chang Y., Li H., Li F., Zou Q., Liu X., Chen X., Cui J. (2024). Inflammation mediates the relationship between diet quality assessed by Healthy Eating Index-2015 and metabolic syndrome. Front. Endocrinol..

[20] Morze J., Danielewicz A., Hoffmann G., Schwingshackl L. (2020). Diet quality as assessed by the Healthy Eating Index, Alternate Healthy Eating Index, Dietary Approaches to Stop Hypertension score, and health outcomes: A second update of a systematic review and meta-analysis of cohort studies. J. Acad. Nutr. Diet..

[21] Taylor R.M., Haslam R.L., Herbert J., Whatnall M.C., Trijsburg L., de Vries J.H.M., Josefsson M.S., Koochek A., Nowicka P., Neuman N. (2024). Diet quality and cardiovascular outcomes: A systematic review and meta-analysis of cohort studies. Nutr. Diet..

[22] Pereira M.A., Jacobs D.R., Van Horn L., Slattery M.L., Kartashov A.I., Ludwig D.S. (2002). Dairy consumption, obesity, and the insulin resistance syndrome in young adults: The CARDIA study. JAMA.

[23] Sumislawski K., Widmer A., Suro R.R., Robles M.E., Lillegard K., Olson D., Koethe J.R., Silver H.J. (2023). Consumption of tree nuts as snacks reduces metabolic syndrome risk in young adults: A randomized trial. Nutrients.

[24] Widmer A., Lillegard K., Wood K., Robles M., Fan R., Ye F., Koethe J.R., Silver H.J. (2025). Consumption of tree nuts as snacks stimulates changes in plasma fatty acid profiles and adipose tissue gene expression in young adults at risk for metabolic syndrome. Clin. Nutr..

[25] Gans K.M., Risica P.M., Wylie-Rosett J., Ross E.M., Strolla L.O., McMurray J., Eaton C.B. (2006). Development and evaluation of the nutrition component of the Rapid Eating and Activity Assessment for Patients (REAP): A new tool for primary care providers. J. Nutr. Educ. Behav..

[26] Segal-Isaacson C.J., Wylie-Rosett J., Gans K.M. (2004). Validation of a short dietary assessment questionnaire: The Rapid Eating and Activity Assessment for Participants short version (REAP-S). Diabetes Educ..

[27] White M.A., Whisenhunt B.L., Williamson D.A., Greenway F.L., Netemeyer R.G. (2002). Development and validation of the food-craving inventory. Obes. Res..

[28] Reedy J., Lerman J.L., Krebs-Smith S.M., Kirkpatrick S.I., Pannucci T.E., Wilson M.M., Subar A.F., Kahle L.L., Tooze J.A. (2018). Evaluation of the Healthy Eating Index-2015. J. Acad. Nutr. Diet..

[29] Krebs-Smith S.M., Pannucci T.E., Subar A.F., Kirkpatrick S.I., Lerman J.L., Tooze J.A., Wilson M.M., Reedy J. (2018). Update of the Healthy Eating Index: HEI-2015. J. Acad. Nutr. Diet..

[30] Rothman K.J. (1990). No adjustments are needed for multiple comparisons. Epidemiology.

[31] Lawton C.L., Delargy H.J., Smith F.C., Hamilton V., Blundell J.E. (1998). A medium-term intervention study on the impact of high- and low-fat snacks varying in sweetness and fat content: Large shifts in daily fat intake but good compensation for daily energy intake. Br. J. Nutr..

[32] Li H., Li X., Yuan S., Jin Y., Lu J. (2018). Nut consumption and risk of metabolic syndrome and overweight/obesity: A meta-analysis of prospective cohort studies and randomized trials. Nutr. Metab..

[33] Shan Z., Rehm C.D., Rogers G., Ruan M., Wang D.D., Hu F.B., Mozaffarian D., Zhang F.F., Bhupathiraju S.N. (2019). Trends in dietary carbohydrate, protein, and fat intake and diet quality among US adults, 1999–2016. JAMA.

[34] Akhlaghi M. (2024). The role of dietary fibers in regulating appetite, an overview of mechanisms and weight consequences. Crit. Rev. Food Sci. Nutr..

[35] Akhlaghi M., Ghobadi S., Zare M., Foshati S. (2020). Effect of nuts on energy intake, hunger, and fullness, a systematic review and meta-analysis of randomized clinical trials. Crit. Rev. Food Sci. Nutr..

[36] Tan S.Y., Mattes R.D. (2013). Appetitive, dietary and health effects of almonds consumed with meals or as snacks: A randomized controlled trial. Eur. J. Clin. Nutr..

[37] Zhang W., Zaved Waise T.M., Toshinai K., Tsuchimochi W., Naznin F., Islam M.N., Tanida R., Sakoda H., Nakazato M. (2020). Functional interaction between ghrelin and GLP-1 regulates feeding through the vagal afferent system. Sci. Rep..

[38] Muller T.D., Nogueiras R., Andermann M.L., Andrews Z.B., Anker S.D., Argente J., Batterham R.L., Benoit S.C., Bowers C.Y., Broglio F. (2015). Ghrelin. Mol. Metab..

[39] Marzullo P., Verti B., Savia G., Walker G.E., Guzzaloni G., Tagliaferri M., Di Blasio A., Liuzzi A. (2004). The relationship between active ghrelin levels and human obesity involves alterations in resting energy expenditure. J. Clin. Endocrinol. Metab..

[40] Chao A.M., Jastreboff A.M., White M.A., Grilo C.M., Sinha R. (2017). Stress, cortisol, and other appetite-related hormones: Prospective prediction of 6-month changes in food cravings and weight. Obesity.

[41] Beauchamp G.K. (2016). Why do we like sweet taste: A bitter tale?. Physiol. Behav..

[42] Jayasinghe S.N., Kruger R., Walsh D.C.I., Cao G., Rivers S., Richter M., Breier B.H. (2017). Is sweet taste perception associated with sweet food liking and intake?. Nutrients.

[43] Ventura A.K., Worobey J. (2013). Early influences on the development of food preferences. Curr. Biol..

[44] Boswell R.G., Kober H. (2016). Food cue reactivity and craving predict eating and weight gain: A meta-analytic review. Obes. Rev..

[45] U.S. Department of Agriculture, U.S. Department of Health and Human Services *Dietary Guidelines for Americans, 2020–2025*, 9th ed.; December 2020. https://www.dietaryguidelines.gov/.

[46] Pashaei K.H.A., Namkhah Z., Sobhani S.R. (2024). Comparison of diet quality indices for predicting metabolic syndrome in Iran: Cross-sectional findings from the persian cohort study. Diabetol. Metab. Syndr..

[47] Konikowska K., Bombala W., Szuba A., Rozanska D., Regulska-Ilow B. (2022). Metabolic syndrome is associated with low diet quality assessed by the Healthy Eating Index-2015 and low concentrations of high-density lipoprotein cholesterol. Biomedicines.

[48] Hosseini-Esfahani F., Daei S., Ildarabadi A., Koochakpoor G., Mirmiran P., Azizi F. (2024). Associations between global diet quality score and risk of metabolic syndrome and its components: Tehran lipid and glucose study. J. Obes. Metab. Syndr..

