# Supplementary material for: Consuming Tree Nuts Daily as Between-Meal Snacks Reduces Food Cravings and Improves Diet Quality in American Young Adults at High Metabolic Syndrome Risk
Source: Nutrients. 2025 Dec 2;17(23):3778. doi: 10.3390/nu17233778 (PMC12694199; doi:10.3390/nu17233778)
Supplement: Supplementary file 1 [file nutrients-17-03778-s001.zip › nutrients-3990994-supplementary.pdf]

nutrients

MDPI

**Table S1: Changes in Micronutrient Intakes Over a Period of 16 Weeks.**

| | TN Snack Group | | | CHO Snack Group | | |
|---|---|---|---|---|---|---|
| | Baseline | End of Study | *p*-value | Baseline | End of Study | *p*-value |
| Vitamin A (IU) | 6516.06 ± 10288.87 | 3842.11 ± 2014.67 | 0.04 | 9302.35 ± 11478.47 | 6969.67 ± 4858.87 | 0.15 |
| Vitamin D (mcg) | 6.73 ± 13.54 | 6.53 ± 13.25 | 0.95 | 5.29 ± 6.35 | 4.50 ± 6.04 | 0.54 |
| Vitamin E (mg) | 9.10 ± 5.92 | 12.55 ± 8.16 | 0.03 | 9.85 ± 7.18 | 9.94 ± 4.18 | 0.95 |
| Vitamin K (mcg) | 141.46 ± 135.61 | 119.55 ± 129.87 | 0.49 | 181.61 ± 187.28 | 144.34 ± 106.23 | 0.25 |
| Vitamin C (mg) | 62.47 ± 55.88 | 57.66 ± 54.93 | 0.68 | 70.05 ± 53.95 | 77.21 ± 54.33 | 0.49 |
| Vitamin B1 (mg) | 1.66 ± 0.69 | 1.74 ± 0.55 | 0.55 | 2.01 ± 0.73 | 1.90 ± 0.84 | 0.49 |
| Vitamin B2 (mg) | 2.07 ± 1.46 | 1.86 ± 0.60 | 0.31 | 2.34 ± 1.03 | 2.40 ± 0.89 | 0.64 |
| Vitamin B3 (mg) | 26.94 ± 19.83 | 24.58 ± 13.12 | 0.48 | 28.41 ± 10.64 | 29.60 ± 12.41 | 0.56 |
| Pantothenic Acid (mg) | 5.14 ± 1.99 | 5.41 ± 2.41 | 0.57 | 5.98 ± 2.53 | 6.08 ± 2.32 | 0.82 |
| Vitamin B6 (mg) | 2.16 ± 1.80 | 2.19 ± 0.97 | 0.92 | 2.23 ± 1.13 | 2.24 ± 0.99 | 0.96 |
| Folate (mg) | 364.00 ± 178.02 | 322.26 ± 158.99 | 0.24 | 441.76 ± 218.63 | 423.43 ± 227.76 | 0.69 |
| Vitamin B12 (mg) | 2.98 ± 1.88 | 3.24 ± 2.45 | 0.60 | 4.11 ± 3.30 | 4.17 ± 2.66 | 0.91 |
| Calcium (mg) | 1092.79 ± 680.04 | 1143.31 ± 591.82 | 0.64 | 1159.03 ± 611.15 | 1143.63 ± 500.28 | 0.88 |
| Phosphorous (mg) | 1268.83 ± 605.69 | 1319.03 ± 470.53 | 0.63 | 1401.66 ± 526.43 | 1403.94 ± 554.10 | 0.98 |
| Magnesium (mg) | 344.25 ± 169.25 | 369.80 ± 109.90 | 0.45 | 364.67 ± 143.42 | 358.67 ± 168.91 | 0.82 |
| Iron (mg) | 13.54 ± 5.74 | 14.06 ± 5.30 | 0.56 | 16.51 ± 7.47 | 15.77 ± 5.43 | 0.52 |
| Zinc (mg) | 10.29 ± 5.40 | 10.97 ± 3.90 | 0.49 | 11.67 ± 6.60 | 12.27 ± 6.09 | 0.59 |
| Copper (mg) | 1.24 ± 0.51 | 1.95 ± 0.56 | <0.001 | 1.34 ± 0.45 | 1.39 ± 0.57 | 0.60 |
| Selenium (mcg) | 124.81 ± 55.46 | 126.50 ± 52.10 | 0.82 | 135.46 ± 50.87 | 138.29 ± 63.47 | 0.78 |
| Sodium (mg) | 3586.21 ± 1298.30 | 2874.90 ± 1230.30 | 0.006 | 4304.70 ± 1986.25 | 4013.98 ± 1154.83 | 0.34 |
| Potassium (mg) | 2421.30 ± 1000.12 | 2520.86 ± 983.40 | 0.63 | 2611.64 ± 895.69 | 2741.97 ± 1090.67 | 0.47 |

*NDSR version 2022 was used to determine micronutrient intakes from 24-hour diet recalls.
